# Applying Precision Public Health to Prevent Preterm Birth

**DOI:** 10.3389/fpubh.2017.00066

**Published:** 2017-04-04

**Authors:** John P. Newnham, Matthew W. Kemp, Scott W. White, Catherine A. Arrese, Roger J. Hart, Jeffrey A. Keelan

**Affiliations:** ^1^School of Women’s and Infants’ Health, The University of Western Australia, Crawley, WA, Australia; ^2^Department of Maternal Fetal Medicine, King Edward Memorial Hospital, Subiaco, WA, Australia

**Keywords:** preterm birth, cervix length, progesterone, smoking, genomics, proteomics, transcriptomics, screening

## Abstract

Preterm birth (PTB) is one of the major health-care challenges of our time. Being born too early is associated with major risks to the child with potential for serious consequences in terms of life-long disability and health-care costs. Discovering how to prevent PTB needs to be one of our greatest priorities. Recent advances have provided hope that a percentage of cases known to be related to risk factors may be amenable to prevention; but the majority of cases remain of unknown cause, and there is little chance of prevention. Applying the principle of precision public health may offer opportunities previously unavailable. Presented in this article are ideas that may improve our abilities in the fields of studying the effects of migration and of populations in transition, public health programs, tobacco control, routine measurement of length of the cervix in mid-pregnancy by ultrasound imaging, prevention of non-medically indicated late PTB, identification of pregnant women for whom treatment of vaginal infection may be of benefit, and screening by genetics and other “omics.” Opening new research in these fields, and viewing these clinical problems through a prism of precision public health, may produce benefits that will affect the lives of large numbers of people.

## Introduction

Preterm birth (PTB) is the single major cause of death in children up to 5 years of age in the developed world (1–3). In low-resource countries, early birth is the second greatest cause of death in young children, second only to pneumonia. Most children born too early will survive and go on to lead a normal and healthy life, but for many there will be life-long disability. Health issues may include neurodevelopmental delay, hearing and visual loss, cerebral palsy, and learning and behavioral problems. The potential impact on individuals, families, and society are considerable (4, 5).

Providing optimal care for very preterm infants in dedicated neonatal intensive care units is vital to minimize any potential for life-long harm, but such care comes at considerable financial cost. The potential costs to society in terms of lost productivity throughout the lifespan may be even greater (6). Considerable benefit has arisen from the discovery in 1972 that administration of corticosteroids to the mother at risk of early birth will halve the rate of death and respiratory distress syndrome in the preterm newborn, but the treatment does not in itself delay the age at birth (7, 8).

Preterm birth is defined as birth before 37 and after 20 completed weeks of gestation. There are many potential pathways to this complication of pregnancy (9). At all gestational ages, and especially the very early ages, inflammation in the pregnant uterus, either due to infectious or non-infectious causes, is commonly associated with preterm deliveries (10). For cases where the age is closer to term, a major concern is medical intervention which at times may not be medically indicated. In other cases, the delivery may be expedited to prevent stillbirth or maternal morbidity, such as for preeclampsia, fetal growth restriction, or diabetes. In half of all cases of PTB, labor commences spontaneously for no known reason or the membranes rupture unexpectedly leading later to the birth. These various phenotypes are diverse and poorly classified, reflecting our incomplete understanding of their various causal pathways and mechanisms.

The impetus to discover strategies by which PTB rates may be lowered has been driven at least in part by advances in neonatal care resulting in more babies surviving at lower gestational ages at birth, but often at high cost in human and financial terms. Starting in the 1960s, much attention was given to developing and refining tocolytic drugs, which are therapeutic agents aiming to inhibit uterine contractions and hence prevent early birth. These drugs may have some usefulness in delaying PTB by hours or a few days, but do not extend pregnancies to gestational ages that will lower the rate of PTB (11).

## Success so FAR

There has been success in lowering PTB rates to some extent in some environments (5). In USA, the rising rates of non-medically indicated late preterm and early-term birth, peaking in 2007, led to the launch of several quality assurance programs aiming to prevent unnecessary early births (6). These programs have resulted in successfully lowering the rates of late PTB in those hospitals and regions that were targeted. In part, as a result of these programs, the rate of PTB in USA fell for 8 years in a row, but has now been reported in late 2016 to have increased again from 9.57 to 9.63% (12).

The first whole of population and whole of geographic region PTB prevention initiative was recently reported for the state of Western Australia (13). Six strategies were applied: administration of progesterone based on prior history of a PTB or the finding of a shortened cervix measured routinely in mid-pregnancy on ultrasound examination, appropriate use of cervical cerclage, avoidance of non-medically indicated induction of labor or Cesarean section, avoidance of exposure to cigarette smoke, judicious use of fertility treatments, and a dedicated PTB prevention clinic at the state’s sole tertiary level perinatal center for referral of cases at highest risk. Implementation involved a state-wide outreach program aiming to ensure that all obstetricians, general practitioners, midwives, and ultrasound imaging specialists had training and expertise in the various aspects of the program. Women and their families were made aware of the strategies through print and social media. The program overall was badged as thewholeninemonths™. After the first full 12 months of implementation, the state-wide rate of PTB had reduced by 7.6% when compared with the years prior to initiation. Statistical modeling estimated that approximately 200 preterm births had been prevented with avoidance of more than 40 in the <32-week gestational age group. Analysis by run charts indicated the rate of late PTB had decreased rapidly, suggesting an effect of educational programs aiming to discourage practitioners and women from unnecessary early intervention. A more delayed effect was observed in reducing births in the 28–31 week category, possibly reflecting use of cervix length screening, administration of progesterone, and surgical cerclage. The benefits extended across the gestational age spectrum from 28–31 weeks onward, although any effect at ages before that time was not statistically significant possibly due to low numbers. An even greater effect in reducing the overall PTB rate was observed within the tertiary level center itself, where awareness among practitioners and pregnant women may have been greater. Together, these results indicate that a comprehensive and multi-faceted geographic-based PTB prevention program in a relatively high resource setting can significantly reduce the rate of PTB using existing knowledge, with an effect of 7–8%.

The magnitude of this reduction in PTB rates recently observed in the Western Australian program is generally consistent with a previous estimate of the effect that could result from effective implementation of known strategies. In an analysis of the potential reduction in preterm births for countries with a very high human development index conducted by the Boston Consulting Group and published in 2013, it was estimated conservatively that the combined impact of implementing known strategies may be a reduction in rate of 5% (14).

We are now left with two great challenges. First is to explore how the array of known strategies can be applied effectively across other population groups. Second is to discover new strategies by which our modest success so far can be expanded. One possibility may be to apply the principles of Precision Public Health to the field of PTB prevention.

## Precision Public Health

Precision public health has arisen from the emerging field of precision medicine (15, 16). Contemporary clinical practice is built upon practice guidelines that are developed and refined by the principles of evidence-based medicine. Typically, such guidelines are applied to individuals with specified symptoms or signs or abnormalities in laboratory or imaging tests. There is much greater potential benefit if the case selection could be refined by tests predicting that a given treatment is likely to be most effective for a given individual with a given disease. As an example, the drug crizotinib has been shown to be much more effective for the treatment of non-small cell lung cancer if the patient has a particular chromosomal translocation involving the gene encoding ALK that drives tumor growth (17).

But there is far more potential to the concept of precision medicine than just “drugs, genes, and disease.” Rather than merely targeting individuals by identifying specific phenotypic or genotypic characteristics, a population approach is possibly the next logical step. Identifying population groups rather than just individuals may yield great benefits. With such an approach, development of precision public health may enable us to benefit the right population at the right time.

Using a population-level approach, the massive amount of data currently being generated from multiple sources could be harnessed for the purpose. Such data collection systems include genomic, transcriptomic and microbiome analysis, and lifestyle characteristics measurable by electronic machines worn or carried by individuals. We also need to expand our capabilities in harnessing data that describe the social and environmental determinants of our health. Clinicians have traditionally incorporated informal information on these determinants into their clinical decision-making, but never before have we had access to data that can provide an accurate description of such factors. We are no longer lacking in information. Our challenge is how to embrace these massive data sources and use the information to identify the right populations at the right time for the right treatment.

**Figure 1:**
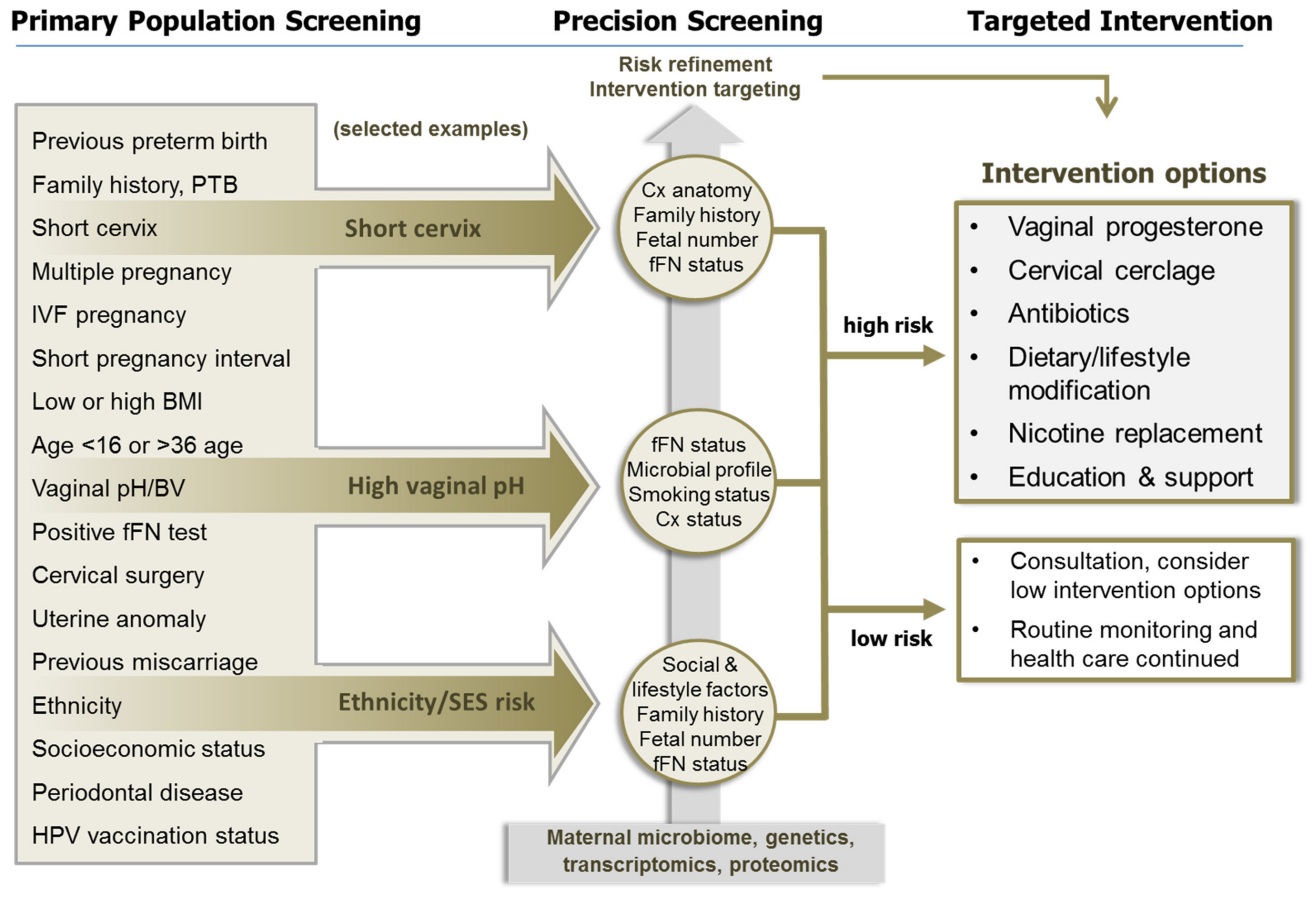
**The application of precision population health principles to preterm birth (PTB) prevention**. Primary population screening using a number of known epidemiological, social, and obstetric factors can identify women with increased risk of PTB but has poor predictive value. In the three examples illustrated, women identified with a specific risk factor are then further evaluated using precision screening, aided by the application of various “omic” technologies, to identify those at greater risk of PTB associated with specific etiologies. This precision risk profile is then used to target treatment and prevention strategies to those who are most likely to benefit. Women who are unlikely to benefit from treatment are also identified through this process. This approach will enhance efficacy, reduce unnecessary interventions, and make optimal use of clinical resources. Abbreviations: BMI, body mass index; BV, bacterial vaginosis; Cx, cervix; fFN, fetal fibronectin; PTB, preterm birth.

The purpose of this article is to explore ways in which the principles of precision public health could be applied to the field of PTB prevention. Different scenarios will be discussed covering some of the strategies that are currently being employed for this task, and we will explore some of the possible avenues by which the principles of precision public health may be incorporated. The concept and examples are illustrated diagrammatically in Figure 1.

## National Differences, Populations in Transition and Health Inequalities

There are many lessons to be learnt from studying the rates of PTB in various nations, how these rates change with time, and the effects of migration.

The PTB rate varies markedly between different countries. In a study of national estimates of PTB rates in 2010 and published in 2013, rates ranged from 5.3 per 100 live births in Latvia to 14.7 per 100 live births in Cyprus (14). Northern European countries had very low rates ranging from 5.5 per 100 live births in Finland to 5.9 in Sweden and 6.0 in Norway. More southern European nations had slightly higher rates with 6.5 in Italy, 6.7 in France, and 8.0 in the Netherlands. In contrast, the rate in USA was 12.0 per 100 live births.

Different migrant subgroups have different reproductive responses to migration (18). Black and Hispanic migrant women are less likely to deliver preterm than US-born Blacks and Hispanics, but the effect does not extend to White and Asian migrants. The duration of residence in the new country also contributes greatly. In a study of migrants to Canada, recent immigrants of less than 5 years had a lower risk of PTB than non-immigrants; but after 15 years or more, the protective effect was reversed and a higher risk of PTB than in non-immigrants was observed. No such effect was found on birth weight relative to gestational age (19). Chinese women living in Jiangsu Province were found to have a much lower rate of PTB than China-born women living in Hong Kong and Western Australia (20). In China-born women living in Western Australia, the ability to be fluent in English and no longer need a translator was associated with a doubling in risk of the pregnancy ending preterm, suggesting that environment was overriding genetics in terms of PTB risk.

The wide variation in PTB rates between countries, and the changes observed with migration, provides strong evidence for environmental contributors and clues to the magnitude of effect that may be amenable to change in any prevention strategy. But what factors associated with migration are operating to decrease and increase PTB rates, and how might this information be exploited in a precision public health approach? Large amounts of data are now available in various government agencies on people as they either reside in their country of origin or migrate. Details are also available on when they moved, at what age, and the circumstances under which they re-located. It seems likely that harnessing these databases would add greatly to our ability to identify women at risk of PTB and enable us to devise public health strategies that may mitigate this risk. Much of the information of course is collected for other purposes and the challenge now is to bridge the gap with the many government and private agencies that generate and control such data bases.

Geographic information systems (GIS) employ sophisticated software and hardware platforms to analyze and collate data on geospatial distribution of disease incidence, risks, and health outcomes (21–23). While the technology and its applications are in their infancy, studies have shown that GIS is a useful modality for guiding public health policy and gaining high precision data on population risks, comorbidities, changes in incidence rates, effectiveness of treatment programs, and socioeconomic factors associated with risk (24–26). As far as we are aware, this approach has not yet been explored for PTB prevention, but there are clearly opportunities to be exploited here for improved and innovative public health PTB prevention initiatives.

A variety of population-based PTB prevention programs are now underway, each targeting the needs of their own communities and aiming to overcome deficiencies that may be contributing to high rates of early birth. In USA, several programs have been launched aiming to overcome health inequalities. Much of this work has resulted from awareness of the very different rates of PTB among the various racial groups. In 2014, the rates ranged from 13.2% in non-Hispanic Black women through to 9.4% in Hispanics and 8.9% in non-Hispanic Whites (27, 28).

In Kentucky, one such program named “Healthy Babies Are Worth The Wait” has focused on improving access to antenatal care by incorporating a range of modifiable factors including group care (6, 28). When compared with surrounding states, there has been evidence of improved rates of PTB.

Central to the high rates of PTB in women of poor socioeconomic and educational standards may be factors operating in their neighborhoods and lifestyles that produce chronic stress and a sense of alienation (6, 28). Living in environments of high crime rates, low wages, lack of employment opportunities, sub-standard services, and feeling excluded from society is associated with higher rates of PTB. Understanding the pathways by which these multiple factors lead to pregnancy complications and other adverse health outcomes will require a much broader view of an individual’s life than we have previously appreciated. Tackling these considerable challenges may be assisted by access to large data bases and an understanding of the importance of the entire life-course in health and disease, as well as gaining a better understanding of how inter-generational effects, such as history of slavery and disempowerment may leave enduring effects on people and their communities. Much work is left to be done, but applying the principles of Precision public health may enable progress that has so far remained elusive.

## Tobacco Exposure and PTB

Exposure to tobacco products, either directly or from environmental sources, is recognized as a significant threat to human health and a significant cause of PTB. The importance of controlling tobacco exposure during pregnancy is underscored by tobacco exposure being designated the single largest preventable risk factor for non-communicable human disease (29). Data published by the World Health Organization show that some 6% of all female and 12% of all male deaths are attributable to tobacco use (30). By 2020, the WHO projects that 7.5 million people will die from direct and indirect exposure to tobacco smoke (30). Data drawn from populations in the United States, Denmark, Sweden, and Canada (i.e., broadly high-income economies) suggest that fewer than 50% of women who smoke cease smoking during pregnancy; Swedish data from 2000 suggest that 13% of women in that country smoked during pregnancy, with smoking persistence more likely in those women with a lower level of educational attainment (31). Accordingly, tobacco control and PTB prevention constitute tightly intertwined and hugely important global public health challenges.

### Tobacco Use and Pregnancy

The greatly elevated risks of lung cancer, chronic respiratory disease, and cardiovascular disease [71%, 42%, and 10% of total incidence, respectively (30)] associated with smoking are well appreciated by both the medical community and the general public; however, the risk of adverse pregnancy outcomes associated with maternal tobacco exposure, and the benefits of smoking cessation prior to or early in pregnancy, are equally profound (32–34). Although protective for preeclampsia (35, 36), smoking during pregnancy is causally associated with fetal growth restriction, placental abruption (37), PTB (38, 39), and sudden infant death syndrome (40). At least one-third of all cases of fetal growth restriction in developed countries is attributable to the effects of maternal tobacco use (41). There are also data to suggest that fetal exposure to many chemicals in tobacco smoke is associated with a host of childhood developmental abnormalities, including subnormal weight gain (42) and neuro-behavioral disorders, such as attention deficit hyperactivity disorder and deficits in auditory and cognitive ability (43).

A 1990 report by the US Surgeon General concluded that “women who stop smoking before pregnancy or during the first 3–4 months of pregnancy reduce their risk of having a low birth weight baby to that of a woman who never smoked” (44). Perhaps, the most important element of this report, in keeping with seminal studies into smoking-related mortality among UK doctors by Doll and colleagues, is that the adverse effects of tobacco exposure on pregnancy are not immutable and can be reduced or even entirely abrogated by timely smoking cessation (45). Numerous subsequent studies, both experimental (46) and epidemiological (47–49), have demonstrated both the significant potential harm to pregnancy and the developing fetus caused by maternal tobacco smoke exposure and, promisingly, the profound benefits to both that may be gained from effective tobacco control.

It is now well established that PTB is a complex syndrome, in many cases likely patient and/or population specific, and a syndrome for which very few preventative interventions exist (9). Of the limited armamentarium presently available to the medical and public health communities, tobacco control is among the most uniformly effective interventions. A meta-analysis of the effects of implementing smoke-free legislation in North America and Europe reported a statistically significant 10% reduction in PTB [1,366,862 cases; −10.4% (95% CI −18.8 to −2.0); *p* = 0.016] (48). Similar benefits have been reported in response to tobacco control measures adopted in Switzerland, with benefits to population pregnancy health correlating with the extent of tobacco control achieved in a particular canton (50). Recent modeling by Levy and colleagues identified that a $2/pack cost increase in cigarette excise tax, combined with cessation programs, health warnings, and public smoking bans would deliver a 33.5% reduction in smoking prevalence among US women aged 15–49 by 2065; relative to maintaining the *status quo*, such policy measures would deliver 227,300 fewer (132,600–302,300) low birth weight infants and 351,000 (137,100–501,300) fewer preterm births over the same period (51).

### Controlling Tobacco Exposure to Prevent PTB *via* Precision Public Health: A Nuanced, Multilevel Approach

Reducing the adverse impact of tobacco exposure on pregnancy outcomes likely requires a combination of national-level initiatives, supported by complementary, culturally, and contextually appropriate programs targeted at specific communities and individual patients. Given the substantial body of data linking tobacco exposure to human disease generally (29, 45), preventive programs based around national public education and legislative control (52) (high levels of tobacco excise tax, comprehensive advertising bans, prohibition of tobacco use in public places, public education campaigns) are clearly warranted and, when well-executed, demonstrate marked reductions in population-level tobacco use coincident with significant reductions in population PTB rates (47, 48, 50). Such programs, targeting both males and females, are important as there is very good evidence that both direct maternal tobacco use and environmental exposure in the home, workplace, or public transport, convey sizeable risks to pregnancy health. In addition to the risks of second-hand smoke, data from the Generation R study in the Netherlands also suggest that, in a number of communities, partner smoking is associated with smoking during pregnancy (53).

Unfortunately, countries employing comprehensive, multi-faceted tobacco control measures at a national level remain in the minority. Many of the countries with sub-optimal tobacco control measures are the same low- and middle-income countries that are home to 80% of the world’s smokers (31) and report some of the highest rates of PTB in the world (2, 4, 54). Even in countries with effective national-level controls, there are marked differences in the effectiveness of national-level tobacco control measures between communities (53, 55), seemingly contingent on a host of socioeconomic factors. In addition to determining maternal tobacco use history and environmental exposure (especially in the home), a precision public health approach to preventing tobacco-associated PTB would likely require assessment of maternal and/or community factors, including (but not limited to), ethnicity, education, poverty, the ability to access social support networks, and the ability to access health-care services.

Ethnicity should be accounted for when designing pregnancy smoking risk assessments and interventions. An analysis of smoking habits in the Netherlands as part of the Generation R study showed significant differences in smoking rates between women of Turkish (43.7%), Dutch (24.1%), and Moroccan (7.0%) ethnicities; moreover, women of Turkish and Moroccan ethnicity were more likely to continue smoking during pregnancy (72.0 and 70.6%, respectively) than women of Dutch ethnicity (58.6%) (53). In South Australia, data collected during the 1990s revealed a marked difference in smoking rates between Aboriginal and non-Aboriginal women (57.8 vs. 24.0%) at their first antenatal visit (56). A similar disparity is evident in more recent data collected in Queensland between 2005 and 2006, with 54% of Indigenous and 19% of non-Indigenous women reporting smoking during pregnancy (55). In the study, adjusted pregnancy outcomes in non-smoking Indigenous and non-Indigenous women were almost equivalent, underscoring the profound impact of smoking on pregnancy and the potential benefits to be gained from effective cessation support (55).

A mother’s level of educational attainment and financial situation are known to be important predictors of peri-conceptual smoking habits and the ability to quit smoking once pregnancy is established (31, 57). Hibbs and colleagues undertook a cross-sectional study of PTB rates among women residing in low-income urban areas in Illinois; the findings of this study showed that impoverished African-American women who smoked exhibited a pattern of “weathering” with advanced maternal age associated with increased rate of PTB [25.2% among 30- to 35-year-old women vs. 17.9% for teenagers; RR = 1.5 (1.1–2.0)]. Interestingly, the authors reported that impoverished White mothers (irrespective of smoking status) and non-smoking African-American mothers did not exhibit a similar pattern of increased rates of PTB with increasing maternal age. The authors concluded that their findings underscore the “potential public health benefit of cigarette smoking cessation programs aimed at the most economically disadvantaged African-American women” (57).

The level of social support and community integration available to a mother is believed to play an important role in pregnancy outcomes and may impact behaviors, such as smoking, during pregnancy. Indeed, smoking has been reported as one means by which women cope with stress in adverse domestic situations, including poverty and disadvantage. In a Turkish study, Ergin et al. reported that young age (<20), low education level, and migrant status were associated with smoking during pregnancy (58). Similarly, in a German cohort, Elsenbruch and colleagues reported that a lack of social support in pregnancy is a key risk-factor for adverse outcomes and that women categorized as having low social support during pregnancy were far more likely (34 vs. 17%) to self-report smoking during the first trimester of pregnancy than women categorized as having high levels of social support (59). These findings mirror earlier findings summarized by Dejin-Karlsson and colleagues, wherein women with strong social support networks are more successful at successfully quitting smoking, and that an absence of support networks is one reason why some pregnant women continue to smoke (60).

Given the relationships between PTB and inadequate antenatal care, economic disadvantage, low-educational attainment, and ethnicity, a number of investigators have recommended the use of community outreach programs or clinics involving multiple disciplines sensitive to the particular needs of the community in question (61). Interventions based on nuanced patient risk assessments that consider individual and community-level factors may thus benefit attempts to reduce the rate of tobacco-associated PTB, and should be considered in any precision public health approach to PTB prevention. Risk assessments could be used to design and deliver targeted, culturally and contextually appropriate interventions at community (i.e., comprehensive anti-smoking programs and cessation support through community, religious, sporting and cultural groups, supported by community leaders) and individual (psychotherapy and pharmacotherapy for smoking cessation, midwife outreach programs, access to social support and mental health services, assisted transport to access health-care services) levels.

## Population-Based Cervix Length Screening

While there are many pathways leading to PTB, some cases may be predicted by measurement of the length of the cervix in mid-pregnancy (62–64). This measurement may be done as a component of the standard mid-pregnancy morphology scan typically conducted between 18 and 24 weeks gestation.

If the cervix appears shortened on trans-abdominal scan, a transvaginal scan is then recommended. For pregnancies where the risk of PTB is perceived to be increased, the standard of practice is generally to proceed to transvaginal scan to measure the cervix length.

The finding of a shortened cervix in mid-pregnancy is predictive of PTB, and there are treatments that reduce that risk, at least for singleton pregnancies. Administration of natural progesterone vaginally as a single evening dose has been shown to nearly halve the risk of PTB in such circumstances. This benefit has been shown in large randomized controlled trials and also by meta-analysis of individual patient data (65–67).

So, we now have strong evidence that the risk of PTB can be reduced dramatically, perhaps by about half, in women found in mid-pregnancy to have a short cervix and then using that information to prescribe natural vaginal progesterone which is a relatively simple and safe treatment. Should the test and its subsequent treatment be applied to the entire population of pregnant women?

In the largest American randomized controlled trial, Hassan and colleagues screened 32,091 women by transvaginal scan to identify 733 with a cervix shortened and measuring between 10 and 20 mm (65). Two hundred thirty-six were randomized to the vaginal progesterone treatment group, 229 to placebo and 268 declined to participate further. The effect was to nearly halve the rate of early PTB in those found to be at risk; the number of births <33 weeks was 21 cases in the treatment group (8.9%) and 36 cases in the placebo group (16.1%). Thus, if the findings of this trial were to be replicated in general clinical practice with no women receiving a placebo and all women participating in the treatment, then screening 32,000 women would identify 2.3% of women requiring treatment and would result in the prevention of 47 cases of births <33 weeks (5).

More recently, the largest randomized controlled trial to have been conducted so far, based in UK, observed a non-significant effect on three primary outcomes—fetal death or birth <34 weeks gestation, a composite of neonatal outcomes, and a standardized cognitive score at 2 years of age (68). There were multiple entry criteria and the treatment consisted of vaginal progesterone from 22–24 to 34 weeks. When the data from the various trials are included in an updated meta-analysis, the beneficial effect of vaginal progesterone treatment for prevention of PTB in women with a singleton pregnancy and shortened cervix in mid-pregnancy remains statistically significant (67).

A cohort study assessing the introduction of routine cervix length screening in mid-pregnancy at a single tertiary level center in Chicago, IL, USA, observed a reduction in preterm births that appeared to be spread across the preterm gestational age spectrum when compared with outcomes prior to introduction of the program (69). The reductions were from 6.7 to 6.0% for births <37 weeks, 1.9 to 1.7% for <34 weeks, and 1.1 to 1.0% for <32 weeks gestation. In this population, the frequency of cervix length less than 25 mm in mid-pregnancy was 0.89%.

But would it be cost-effective to introduce this protocol into clinical practice across the entire population? In a USA-based decision analysis model of a single cervix length measurement in mid-pregnancy compared with no such measurement, with treatment with vaginal progesterone if the cervix were found to be shortened, it was estimated the program would save $12 million/year and gain 424 quality-adjusted life years (70).

A more recent USA-based analysis asked if risk-based screening would be more or less effective than universal screening of all pregnancies (71). Results of the decision analytic model indicated that both risk-based and universal screening would be more cost-effective than no screening. Of the two approaches, universal screening of cervix length measurement of all pregnancies was superior to risk-based screening and would result in a higher cost-effectiveness ratio.

Despite the evidence presented above, there remain as many unknowns as knowns. What is the prevalence of shortened cervix in mid-pregnancy in the various populations of the world? Even within the USA there is considerable variation, ranging from 0.89% <25 mm in the Chicago study (69) to 2.3% <20 mm in the multicentered trial reported by Hassan and colleagues (65). Regions such as northern Europe where the rate of PTB is much lower are likely to have even lower prevalence of shortened cervix in mid-pregnancy. Further, we have few data describing the phenotypic and genotypic variables that influence mid-pregnancy cervix length and the response to treatment, and we have little understanding how to monitor and manage cases undergoing treatment and the drivers of treatment success and failure. This is a key issue in the context of precision public health (Figure 1). Interestingly, a small USA study found that progesterone and cerclage therapies were only effective in women with biomarkers of inflammation in the amniotic fluid and that without inflammation the risk of PTB was actually increased (72). This preliminary evidence suggests that the inclusion of additional screening parameters, such as inflammation, might improve precision and response to targeted therapies.

To achieve improvements in precision, we need to progress beyond case studies and randomized controlled trials. In the first instance, we need sound and reliable population-based data. Most high resource nations have perinatal collection systems collecting basic information on demographics, the birth, and the newborn. To fully understand and maximize the benefit from mid-pregnancy cervix length measurement and treatment, such data collection systems will require much greater complexity and capability. The expansion will be complicated by the fact that many health-care systems are fragmented, with antenatal investigations and treatments often being dislocated from hospital-based birthing processes. Prevention of PTB is of great benefit to individuals and the community, and development of cost-effective models of health-care delivery using this intervention should be entirely feasible. At this time, it would seem reasonable to expect that the most effective solution will come from population-based screening, but followed by further precision analysis to best understand the most appropriate treatment for each case and the manner by which that treatment should then be monitored for effectiveness.

## Avoidance of Non-Medically Indicated Late Preterm and Early-Term Birth

The rates of late preterm, and early-term, birth have been increasing over recent decades in many countries (73, 74). These trends have been underpinned by a general assumption held by many people that birth close to term will not be associated with any enduring compromise for the offspring. A wealth of data, from multiple societies, now suggest otherwise.

Being born late preterm, defined as birth between 34 and 36 weeks and 6 days gestation, places the infant at risk of neonatal and childhood consequences (75). For the neonate, complications may include the need to be admitted to a neonatal intensive special care unit, and special support to maintain respiratory function, temperature control, prevention of infection, and maintenance of normal glucose levels, and much more (75, 76). For the child, there are increased risks of death, re-admission to hospital, cerebral palsy, developmental delay, and behavioral and learning problems at school age (77, 78). In recent years, the findings of potential compromise from prematurity for the child have been extended to birth in the early-term period, ranging from 37 weeks and 0 days to 38 weeks and 6 days.

For those cases in which early birth is required for maternal or fetal reasons, the benefits may outweigh any risks of prematurity. But, we now have strong evidence that there are many cases where such a benefit is not the case, and steps need to be taken to ensure that any elective early births can be fully justified.

Population-based study of the factors involved in rising rates of early births has shown clear demographic differences (2, 14, 79). In a USA study of early births between 1992 and 2002, the major increase in rate was observed in non-Hispanic White births (80). During the decade of study, rates of early births in Hispanic and Black women had remained relatively constant. The factors underpinning these observations are uncertain, but suggest that socioeconomic factors are involved and that both medical and patient contributions need to be considered.

Reducing the rate of early intervention is particularly challenging, but progress has been made by some health-care systems. In a study of 27 health-care facilities in USA, each organization was invited to choose one of three protocols to reduce their rate of non-medically indicated late preterm and early-term birth (81). The options ranged from just education of staff through to complete prohibition of early birth. The gestational age below which non-medically indicated birth was to be discouraged was 39 completed weeks. Outcome data revealed that the most interventionist protocols produced the greatest benefit, with significant reductions in early birth rates and admissions to neonatal intensive care units. The still birth rate did not change during the time of the study. Education alone did not improve outcomes, but of importance, the program only involved the medical staff and did not include education of other health-care practitioners or the patients themselves.

At a national level, there has been considerable public advocacy across USA led by the March of Dimes though a campaign called “Healthy Babies are Worth the Wait,” coupled with quality improvement initiatives (6). These programs have been aimed primarily at health-care providers and pregnant women to discourage unnecessary early intervention. The results of these and other programs suggest that the rates of non-medically indicated late preterm and early-term birth rates can be reduced, but there are many confounding factors.

First, the effect is entirely dependent on the extent to which unnecessary early intervention is prevalent in a particular health-care environment. Further, it is clear that individual health-care practitioners and their pregnant patients actively choose to make such decisions. Understanding when preventative policies should be introduced requires detailed understanding of not just health-care outcomes but also the practices of individual practitioners and the attitudes of women and families who access such care.

Second, are the health-care workforce implications of discouraging elective birth before 39 weeks gestation. Such a policy will inevitably increase the number of cases of spontaneous labor, and there are clear implications for the work–life balance of busy practitioners, especially those in solo or small group practices. In a secondary analysis of a randomized controlled trial in Denmark comparing planned elective Cesarean births at 38 or 39 weeks gestation, delaying the surgery by 1 week resulted in a 60% increase in unscheduled Cesarean sections and a 70% increase in births out-of-hours (82). Imposing health-care guidelines that potentially compromise the daily activities of practitioners and hospitals requires considerable justification and a thorough understanding of the issues involved in each health-care environment.

Third, is the potential for stillbirth or fetal compromise by delaying birth. There is no convincing evidence at a population level that earlier delivery is associated with lesser rates of stillbirth but it is logical to assume that a background risk of fetal death must remain present by continuing the pregnancy, even in the absence of any known risk factors. Any such risk would need to be weighed against any potential risk of death or morbidity resulting from late preterm or early-term birth. Reassuringly, the American trial of three management options in 27 hospitals significantly reduced early birth rates and there was no evidence of any increase in risk to the child. In the Danish RCT of elective Cesarean delivery at 38 or 39 weeks gestation, there was a small reduction in rate of delivery to the NICU in the delayed delivery group, but no other signs of danger (82, 83).

It is clear, therefore, that the challenge to prevent non-medically indicated late preterm/early-term births is scientifically justified, but confounded by regional differences, possible benefits and risks to the child by continuing the pregnancy, and potential adverse effects on the practitioners and their health-care facilities. For each health-care environment, detailed and ongoing data availability and analysis are vital if preventative strategies are to be introduced and be maintained. So far, in USA and Western Australia, there has been success in lowering the rates of late preterm/early-term births, but continuing success and translation into other environments where the baseline PTB rates are already lower may be more challenging. The principles of precision public health may offer the solution to this major problem. By applying precision analysis using factors such as genetic predisposition, prior history, and lifestyle variables, we may better understand which pregnancies can safely be left until after 39 weeks gestation and which cases require earlier intervention.

## Prevention of Infection-Driven PTB

It is well established that microbial infection of the extra-placental membranes, amniotic cavity, and fetus is an important driver of PTB, particularly in the deliveries at the earliest gestational ages (9, 84, 85). Animal models and clinical studies demonstrate a causal relationship between infection and PTB, while the immune-pathophysiological pathways responsible for triggering preterm labor in response to infection have been studied, replicated and characterized in a variety of models (86–88). The most common pathway *via* which bacteria trigger PTB is the so-called ascending infection route: microorganisms residing in cervico-vaginal fluid in pregnancy ascend through the cervical barrier and colonize the fetal membranes, passing through in some cases to colonize the amniotic fluid and from there infect the fetus (86, 89). The severity of the infection and the ensuing inflammatory response determines, to a large part, the obstetric and neonatal outcomes, including the timing and onset of preterm labor, the effectiveness of tocolytic therapy, and the risk of serious neonatal morbidities.

While the majority of preterm deliveries occur in the 32- to 36-week period, the costs and risks of serious perinatal morbidities are highest in deliveries <32-week gestation, the majority of which are infection-associated (9, 84, 85). Hence, preventing PTB as a result of intrauterine infection has the largest potential gains in terms of reducing major morbidity and death and reducing perinatal and lifetime health-care costs (90, 91).

Unfortunately, identifying women at risk of infection-driven PTB and treating them to prevent the infection is challenging, both from an individual patient and public health perspective (92–94). Gestational tissues exposed at different times in pregnancy to different bacteria exhibit variable immune responses depending on dose, duration, distribution, maternal and fetal genetics, ethnicity, lifestyle, and anatomical factors (such as previous cervical surgery). This level of heterogeneity makes identification and risk prediction particularly difficult. There are several well-documented approaches which can identify women at increased risk of infection-related PTB, yet their prognostic value is generally too weak to alter clinical decision-making and treatment. Many of the trials of prophylactic antibiotic therapy given to pregnant women to prevent PTB have failed to employ robust inclusion criteria or delivered poorly effective antibiotic regimens (95, 96). Identifying women who will benefit from treatment is a key requirement for primary prevention: prescribing antibiotics to large numbers of women in pregnancy in order to prevent PTB in a small percentage of recipients is not justifiable in light of the emerging recognition of the potential developmental effects of disrupting the maternal and neonatal microbiomes with antibiotics in pregnancy (97–100).

To date, primary prevention research has focused on identifying and treating women with abnormal vaginal microbiota in early pregnancy prior to the onset of preterm labor (101, 102). This strategy is based on the assumption that women with vaginal dysbiosis have increased risk of ascending infection and that antibiotic treatment will eradicate the pathogens, prevent infection, and thus prevent PTB. The odds ratio of women delivering preterm with bacterial vaginosis (BV) or aerobic vaginitis (AV) is approximately 2–7, and even higher if diagnosed before 16-week gestation (103–105). The presence of *Ureaplasma* in the vagina is also associated with an approximately twofold increased risk of PTB (106–108). However, due to its high prevalence in pregnant women (~50%) (109), detection of *Ureaplasma* alone is not sufficiently diagnostic to warrant prophylactic treatment. The prognostic significance of the presence of specific *Ureaplasma* serovars is currently under investigation (109).

A large number of trials have employed standard microbiological or clinical approaches to identify women with vaginal dysbiosis and treated them with antibiotics (with or without probiotics) to prevent PTB (84, 110, 111). Many of these studies failed to significantly lower the rate of PTB (111–114), although a few studies employing clindamycin treatment before 22 weeks of pregnancy have shown significant maternal and neonatal benefits (95, 96). The negative findings are, in part, due to lack of effective antimicrobial interventions (95) and the failure to treat the dysbiosis or address recurrence (115–117). However, the major impediment to therapeutic progress is the poor prognostic precision of current identification methods. Most women with vaginal dysbiosis or urogenital tract pathogens do not deliver preterm, and our ability to identify those at sufficiently high risk to warrant treatment (OR > 10) is poor (102). Primary prevention studies have focused on defining clinical vaginal microbial disorders and recruiting patients based on these definitions, rather than identifying subgroups of women who are at particularly high risk of delivering preterm as a result of microbial profile and/or other risk factors and targeting them for appropriate and effective treatment. Currently, although rapid molecular tests for diagnosing BV and AV are being developed (118, 119), we lack an accurate, rapid, and affordable test to identify women at high risk of infection-related PTB (120, 121).

Nevertheless, despite these uncertainties and qualifications, there is encouraging evidence that public health screening for BV or other forms of abnormal vaginal microbiota using traditional techniques can be an effective primary prevention strategy in reducing PTB rates. A recently published Austrian study, implemented following the results of a randomized clinical trial, retrospectively analyzed the pregnancy outcome data of over 17,000 women at high risk of PTB (based on general, family, and obstetric risk factors) across a 10-year timespan following introduction of a voluntary antenatal infection “screen and treat” program (122). All women received standard antenatal care; 49.5% entered the screen and treat program, which consisted of testing of vaginal swabs at 10- to 16-week gestation for detection of BV and presence of *Candida* spp. or *Trichomonas vaginalis*, followed by antimicrobial treatment with either clindamycin, clotrimazole, or metronidazole as appropriate. Recurrent infections were retreated and women with BV were given probiotics after treatment to prevent recurrence. Women in the treated group had a significantly lower rate of stillbirth (0.4 vs. 2.0%), miscarriage (0.5 vs. 1.4%), PTB <37 weeks (9.7 vs. 22.3%), and PTB <32 weeks gestation (1.9 vs. 8.3%). The effect of the program on the rates of early/extreme PTB was particularly impressive, with a more than 77% reduction observed; this is consistent with the known role of intrauterine infection in the majority of deliveries <32 weeks. The major weakness in this study is its retrospective, non-randomized design, although confounding is minimal and unlikely to alter the findings (122).

These data are similar to the achievements of an earlier public health program implemented in Germany in late 1997 (123). The program consisted of a free self-test vaginal pH kit offered to 2,722 women in obstetric care (>12-week gestation), with optional follow-up with obstetricians if the test was positive. Elevated vaginal fluid pH is a weak surrogate marker of BV and AV, typically associated with a lack of *Lactobacillus* spp. Treatment with antibiotics (clindamycin) was indicated if clinical symptoms of BV were present following obstetric examination. The program resulted in much lower rates of PTB <32 weeks in the women who participated (14% of the cohort) compared to women under the same care who did not engage in the pH testing program (0.3 vs. 4.1%). Subsequently, a large prospective trial was conducted, enrolling 8,000 women in the state of Thuringia over a 6-month period. Women who self-tested their vaginal pH and sought medical treatment based on the result (8% of the cohort) had lower rates of PTB at <32 weeks (0.3 vs. 1.6%) and at <37 weeks of gestation (5.3 vs. 8.5%). After discontinuation of the program, PTB rates returned to historical levels in the state (123).

In order to obtain greater precision, we need to refine our ability to identify women at high risk of infection-driven PTB (OR > 10) and target them with appropriate follow-up and treatment. Many of the bacteria found in the amniotic cavity of preterm deliveries are normal commensals of the urogenital tract (124, 125), so vaginal microbiological profiling alone is unlikely to have a high positive predictive value. It remains to be determined whether more refined and selective molecular techniques may help to improve diagnostic discrimination (109). It is likely that a combination of clinical risk factors (e.g., prior PTB, cervical imaging, and abnormality detection), high-resolution microbial profiling (possibly including bacterial strain identification), and immunological/inflammatory biomarker assessment will be needed, in combination with a highly effective antimicrobial regimen (97, 126), to enable a truly effective maternal “screen and treat” program to achieve the desired level of precision and effectiveness required for a primary prevention public health program (127–133).

## “Omics” and Precision Public Health

### Identification of At-Risk Individuals: Genomic Approach

The contribution of genomic variation to the etiology of PTB is thought to be in the order of 40% by twin and family studies (134). Women who were born preterm, or who have close family members with a PTB, have a significantly higher risk of PTB. Rates of prematurity are influenced by ethnicity (135): studies have shown that the rates of PTB in African-Americans is significantly higher than other racial groups in similar socioeconomic settings, and that women married to African-American men have higher prevalence of PTB. Determining the precise nature of the genomic variants responsible for determining risk of PTB is hampered by the complex biology of preterm labor (135). From an evolutionary perspective, PTB is detrimental to the survival of the species, and there are multiple levels of redundancy in the biological process of labor initiation, which combine to reduce the incidence of PTB. The genetic basis of PTB is, therefore, unlikely to be monogenic. Rather, in all but the most extreme of phenotypes, multiple changes with gene pathways are required to overcome physiological redundancy and culminate in PTB.

The advantage of genome-based screening tests is that they may be applied prior to pregnancy and allow ample time to initiate primary prevention, rather than attempting to slow or reverse the premature activation of parturition which leads to spontaneous preterm labor. Many gene-targeted analyses and genome-wide association studies (GWAS) have been carried out in an attempt to identify genetic variants associated with PTB (135). Sheikh et al., in a recent review of the literature, identified 119 candidate genes with SNPs that had potential association with PTB in an evaluation of 92 different studies. Many studies have found association between SNPs in parturition-associated genes such as the progesterone receptor, oxytocin receptor, relaxin, the prostaglandin EP3 receptor, and the CRH receptor 1, although the level of risk associated with these polymorphisms is not sufficiently high to be useful clinically (136). Other target genes have yielded more promising SNPs, in particular heat-shock protein 47 (SERPINH1), which is involved in the maturation of collagen molecules and is enriched in African and African-American populations. Polymorphism in other tissue remodeling-related genes like metallopeptidase inhibitor-1 and 2, COL1A2, COL5A2, and COL5A1 also significantly increase the risk of PTB (136). Overall, many of the SNPs significantly associated with PTB are related in some way to inflammation. Lack of replication and population-based heterogeneity remain major hurdles to be overcome.

Identification of combinations of multiple subtle genomic contributors are now feasible with advances in high-throughput genomic sequencing and bioinformatics. In a recent study of women with 2–3 generations of PTB using a meta-genomic, bi-clustering algorithm, Uzun et al. (137) identified variations in 33 genes within five genetic pathways associated with altered PTB risk. Brubaker et al. employed protein network analysis with tissue-specific gene expression data to identify functionally important candidate genes that would be overlooked by standard GWAS techniques. Their analysis identified significant sub-networks and genes not previously associated with PTB, including sub-networks associated with inflammation, muscle function, and ion channels (138). It is likely that extensions of such work will ultimately permit the characterization of an individual’s overall genomic risk of PTB as a screening test to identify those at risk, with the molecular consequences of the variation being used to guide preventive interventions.

Further advances are likely to originate from advances in the use of phenotypic data from rare genetic diseases to identify variants associated with common pathways shared by multiple disorders (139). To this end, the recently expanded Human Phenotype Ontology (www.http://human-phenotype-ontology.github.io) now contains 250,000 phenotypic annotations for over 10,000 rare and common diseases (140, 141), which can be used to examine the phenotypic overlap among common diseases with shared risk alleles or those linked by genomic location. Other databases and platforms have been developed to allow accurate assessment of the causal relationship between genetic variants and phenotype; these are becoming critical tools in clinical genetic diagnostics (140), for comparing phenotypes between patient cohorts (142) and for identifying new disease genes *via* the linkage of novel variants with well-defined phenotypes (141, 143). Application of such approaches to understanding the genetic causes of PTB and identifying populations at risk remains to be explored. However, several examples have already been identified. Insights may be expected from analyzing the links between PTB and Prader–Willi Syndrome (144, 145) or by studying Beckwith–Wiedemann Syndrome, which is associated with increased rates of PTB, gestational diabetes, polyhydramnios, and intrauterine bleeding (146). Advances in this area may lead to improved knowledge regarding genetic variants and pathways to PTB which can be exploited to enhance screening programs and develop and target interventions.

### Identification of At-Risk Individuals: Transcriptomic Approach

Transcriptomic methods assess RNA in tissue and quantify the extent to which genes are functioning, rather than inferring variations in function related to variations in sequence. By examining expression of genes related to inflammation, for example, one can find evidence of inflammation prior to the development of clinical manifestations. In PTB, a screening test could be developed to identify individuals with premature activation of parturition at a stage where treatment can reverse such activation prior to the tipping point to inevitable preterm labor (147).

As was the case for genomic methods, alterations in the transcriptome in the lead up to PTB are likely to arise from multiple pathways. Heng et al. (148) described a method considering the expression levels of multiple genes from multiple pathways in maternal whole blood and their relationship to subsequent PTB. Applied to asymptomatic pregnant women at 28-week gestation and including clinical factors, their model predicted PTB with sensitivity of 65%, specificity of 88%, and false positive rate of 11%; their birth cohort had been enriched, with a PTB rate of 31%. This method requires further validation in average-risk populations before being considered for clinical application. Alternative methods may employ samples other than maternal blood such as cervico-vaginal fluid, or analysis of micro RNAs and other related molecules which may give insights into placental pathologies and associated risk factors. With refinement, this approach could be useful in the identification of the woman heading toward PTB in whom interventions could arrest this course and allow safe delivery at term.

### Identification of At-Risk Individuals: Proteomic Approach

As transcriptomics looks at the actual expression of genes rather than the sequence, proteomics goes a step further in examining the protein end products of gene function, giving insight into the physiological alterations related to gene sequence and expression. High-throughput proteomic assessments allow the identification of differentially produced proteins in association with clinically relevant phenotypes. From a PTB perspective, proteomic variation may herald early delivery prior to the development of clinically apparent symptoms.

The most commonly employed protein assessment is fetal fibronectin in women who present with symptoms suggestive of preterm labor. This protein is found in greater quantities in the cervico-vaginal fluid in women who will deliver preterm than in those women whose pregnancy will continue to term (149). Quantitative or qualitative assessment of fetal fibronectin levels permit the rationalization of therapies aimed at delaying delivery or reducing the adverse sequelae of prematurity.

A natural extension of the use of fetal fibronectin testing to stratify risk of PTB in symptomatic women is its application to asymptomatic women. To date, there is little evidence to support the adoption of such screening into routine clinical practice in low-risk pregnant women (150). It may have greater utility in the screening of women with other established risk factors (151–153).

Kim et al. (154) assessed the utility of amniotic fluid MMP-8 as a screening test for subsequent PTB in women undergoing diagnostic amniocentesis in the mid-trimester. The test was highly specific (100%) for subsequent PTB, albeit with relatively low sensitivity (42%). The major limitation of this approach is the invasive nature of the test which is unlikely to be acceptable to the majority of women due to the low but significant risk of pregnancy loss. Several other studies of amniotic fluid proteome have been carried out, with a number of candidate biomarkers identified for predicting PTB and neonatal adverse outcomes secondary to intrauterine inflammation (155, 156), but these have not been clinically exploited (157).

A more acceptable, less invasive testing strategy based on analysis of cervico-vaginal fluids may have greater prognostic potential and acceptance. Gravett et al. (158) undertook a proteomic study of the cervico-vaginal fluid in a non-human primate model of iatrogenic intra-amniotic infection, a major contributor to PTB, with the aim of identifying a non-invasive biomarker for this condition. Twenty-six proteins were found to be differentially expressed in the presence of intra-amniotic infection compared to controls, with a preponderance of proteins involved in inflammatory regulation. Of these, IGFBP1 was increased 16-fold in the presence of intra-amniotic infection, and this is a potential biomarker for clinical application. Many other studies have investigated levels of inflammatory cytokines and proteins in cervico-vaginal fluids, and candidate proteins such as IL-6 have been consistently identified (159, 160), but these have not yielded biomarkers with sufficient prognostic utility to be useful clinically. Georgiou and colleagues employed proteomic analysis of cervico-vaginal fluid samples in at-risk asymptomatic women to identify candidate biomarkers of impending preterm delivery (161). They found that thioredoxin and interleukin 1 receptor antagonist levels were significantly reduced up to 90 days prior to preterm labor compared with women who delivered at term. Both proteins had a positive predictive value of >72% and negative predictive value of >95%. The prognostic value of these biomarkers has yet to be demonstrated in independent studies and populations; however, and to date, proteomic approaches have not yet yielded clinically useful tests that have been commercialized and widely adopted (162).

## Precision Refinement Using New Technologies

“Omic” approaches may be suitable for both primary screening, as well as precision refinement in women previously identified as being at risk of PTB by other screening modalities (Figure 1). Gene–environment interactions underlie almost all responses of complex organisms to external stimuli. This principle is the basis of pharmacogenomics, whereby an individual’s genetic susceptibility to the effects of a drug determines whether or not that drug is used. However, this may be used on a larger scale than is currently employed in order to direct interventions in those who screen at increased risk of PTB. For example, women who are genetically more likely to succeed in smoking cessation with nicotine replacement therapy may be offered this intervention, while those genetically likely to fail may avoid the potential adverse outcomes of this therapy (163).

As high-throughput genomic sequencing technology becomes more affordable and rapid and point-of-care devices are developed, future technological advances may provide exciting new avenues for further refinement of risk. Other risk factors likely to be amenable to “omic” precision refinement include bacterial dysbiosis, previous inflammatory and infection-related PTB, as well as cervical dysfunction (Figure 1).

Other technological advances are likely to be able to be exploited in the near future to gain greater precision in PTB prevention strategies. Wearable mobile sensor technologies have been developed for a number of applications requiring real-time monitoring of physiological parameters that allow monitoring of health status/responses and identification of individuals at risk. Examples include remote monitoring of the elderly after transfer to a community care setting (164, 165). Sophisticated systems have been developed and trialed for measuring multiple physiological parameters (166), and it could be envisaged that such systems could be adapted and used in high-risk women to identify changes in uterine activity, for example, suggestive of early onset of labor (164).

In addition, E-registries and web-based surveillance systems are exciting developments in health information systems that have applications in monitoring maternal health trends and outcomes together with changes in population characteristics and risks (167, 168). A simple example that illustrates the potential is the use of a mobile SMS-based system for monitoring maternal health in low-resource settings (169).

## Author Contributions

JN: first draft of introduction, populations in transition, cervix length screening and non-medically indicated preterm birth, and responsible for overall manuscript. MK: first draft of smoking section and review of entire manuscript. SW: first draft of genetics and -omic section and review of entire manuscript. CA: coordination of authors and review and submission of manuscript. RH: contributed to writing of overall manuscript and review. JK: first draft of infection-associated preterm birth, preparation of figure, and review of overall manuscript.

## Conflict of Interest Statement

The authors declare that the research was conducted in the absence of any commercial or financial relationships that could be construed as a potential conflict of interest. The reviewer, GB, declared a shared affiliation, though no other collaboration, with the authors to the handling Editor, who ensured that the process nevertheless met the standards of a fair and objective review.
